# Molecular Epidemiology of Carbapenem-Resistant *K. pneumoniae* Clinical Isolates from the Adult Patients with Comorbidities in a Tertiary Hospital, Southern Saudi Arabia

**DOI:** 10.3390/antibiotics11121697

**Published:** 2022-11-25

**Authors:** Abdullah M. Alshahrani, Mutasim E. Ibrahim, Ahmed K. Aldossary, Mushabab A. Alghamdi, Omar B. Ahmed, Aref A. Bin Abdulhak

**Affiliations:** 1 Department of Family Medicine, College of Medicine, University of Bisha, Bisha 67614, Saudi Arabia; 2 Department of Basic Medical Sciences (Microbiology Unit), College of Medicine, University of Bisha, Bisha 67614, Saudi Arabia; 3 Department of Internal Medicine, College of Medicine, University of Bisha, Bisha 67614, Saudi Arabia; 4 Department of Environmental and Health Research, The Custodian of the Two Holy Mosques Institute for Hajj and Umrah Research, Umm Al-Qura University, Makkah 24382, Saudi Arabia; 5 Division of Cardiovascular Medicine, Department of Internal Medicine, University of Iowa Carver College of Medicine, Iowa City, IA 52240, USA

**Keywords:** *Klebsiella pneumoniae*, carbapenemase genes, comorbidities, Saudi Arabia

## Abstract

Hospitalized patients are likely to have chronic illnesses and are at an increased risk of mortality due to infection caused by MDR bacteria. We aimed to identify carbapenem-resistant genes carrying *Klebsiella pneumoniae* (*K. pneumoniae*) isolates and their risk factors recovered from adult patients with comorbidities. A cross-sectional study was carried out between April 2021 and December 2021 at King Abdullah Hospital (KAH) in Bisha province, Saudi Arabia. Seventy-one multi-drug resistant *K. pneumoniae* recovered from clinical samples and screened for carbapenemase genes of *bla*OXA-48-like, *bla*NDM-1, *bla*KPC, *bla*VIM, and *bla*IMP. Of 71 MDR *K. pneumoniae* examined, 47 (66.2%) isolates harbored various carbapenemase genes. The most prevalent single resistance gene was *bla*OXA-48-like (62.5%; n = 25), and 33.3% of them were recovered from sputum isolates. The *bla*NDM-1 gene was detected in 12 (30.0%) isolates, and eight of them were recovered from urine (n = 4) and blood (n = 4). Two (5.0%) single *bla*KPC genes were recovered from the sputum (n = 1) and blood (n = 1) isolates. In contrast, no *bla*IMP- and *bla*VIM-carrying isolates were detected. The co-existence of two resistance genes between *bla*OXA-48-like and *bla*NDM-1 was found in six strains, whereas only one strain was found to be produced in the three genes of *bla*NDM-1, *bla*KPC, and *bla*OXA-48-like. There were statistically significant associations between the presence of carbapenem-gene-carrying *K. pneumoniae* and patients’ gender (χ2(1) = 5.94, *p* = 0.015), intensive care unit admission (χ2(1) = 7.649, *p* = 0.002), and chronic obstructive pulmonary disease (χ2(1) = 4.851, *p* = 0.028). The study highlighted the existence of carbapenemase-producing *K. pneumoniae*, particularly *bla*OXA-48-like and *bla*NDM-1, in patients with comorbidities. Our findings emphasize the importance of the molecular characterization of resistance-determinant-carrying bacterial pathogens as a part of infection control and prevention in hospital settings.

## 1. Introduction

*Klebsiella pneumoniae* (*K. pneumoniae*) has emerged as a major human pathogen implicated in infections in hospital settings globally [1]. The treatment of infections caused by *K. pneumoniae* has been a significant threat due to their capability to acquire rapid mechanisms of various antibiotic resistance traits to the several groups of antimicrobial agents [2]. The multidrug-resistant (MDR) trait in *K. pneumoniae* has been recognized in healthcare settings as a cause of high morbidity and mortality among patients with severe infections [3]. Such MDR strains carry a wide range of antimicrobial resistance gene elements that restrict the options for treating *K. pneumoniae* infection [1].

Carbapenems are effective and reliable β-lactam antibiotics often used as the last option for treating severe infections caused by MDR Gram-negative bacteria, including *K. pneumoniae* [4]. However, the increasing use of carbapenems has resulted in the emergence of carbapenem-hydrolyzing β-lactamases (carbapenemases) as a common mechanism of resistance to carbapenem agents [5]. Among the *Enterobacteriaceae* group, clinically significant carbapenemases are the Ambler molecular class A (*bla*KPC), class B (*bla*VIM, *bla*IMP, *bla*NDM-1), and class D (*bla*OXA-48-like) types. These types are commonly found in *K. pneumoniae* and are frequently associated with serious nosocomial infections and outbreaks [5]. 

The genetic diversity and burden of carbapenem resistance in *K. pneumoniae* have been studied in Saudi Arabia. A previous study among hospitalized patients in Saudi Arabia indicated that *bla*OXA-48-like and *bla*NDM-1 are the dominant carbapenemases in *Enterobacteriaceae*, including *K. pneumoniae* [6]. A study in a tertiary care hospital in Riyadh capital reported an outbreak of multi-drug carbapenem-resistant *K. pneumoniae* infection carrying the *bla*OXA-48-like gene [7]. A more recent study at four main tertiary care hospitals of Makkah in the western region of Saudi Arabia reported the emergence of MDR *K. pneumoniae* co-harboring *bla*KPC, *bla*NDM-1, and *bla*OXA-48-like genes [8]. Due to increased carbapenemase-producing bacteria in hospital settings, understanding the resistance mechanisms will offer valuable insights into their management.

However, there are still knowledge gaps and a lack of data in many hospitals in Saudi Arabia regarding carbapenemase-producing bacterial pathogens. This limitation is a significant health concern in hospital settings, as treating patients with nosocomial infections can be associated with high antimicrobial resistance patterns [9]. In addition, hospitalized patients are likely to have chronic illnesses and are at an increased risk of mortality due to infection caused by MDR bacteria [10,11]. A recent study found that infection caused by carbapenem-resistant *K. pneumoniae*, when associated with comorbidities, can lead to a high mortality rate [11]. Furthermore, research evidence indicated that patients with comorbidities have longer exposure time in hospitals, tend to be hospitalized, and are under prolonged antibiotic therapy, which are relevant risk factors for carbapenem-resistant *K. pneumoniae* infection [11,12]. Herein, we characterize the molecular epidemiology of carbapenem-resistant genes carrying *K. pneumoniae* isolated from adult patients with comorbidities. We also aimed to identify predictors of carbapenemase gene acquisition in this population. 

## 2. Materials and Methods

A cross-sectional study was carried out between April 2021 and December 2021 at King Abdullah Hospital (KAH) in Bisha province, Saudi Arabia. This hospital is a referral hospital with 400 beds, including various specialties, serving various patient groups, and covering most populations of different areas in Bisha province and surrounding areas. Adult patients more than 22 years old with diverse chronic comorbidities at the KAH were recruited in the study. Information about patients’ demographic characteristics, detailed records of clinical features, and types of chronic illness present were collected from patient files and hospital databases without violating patient identities. Ethical approval was obtained from Research Ethics Local Committee at the College of Medicine, University of Bisha, Saudi Arabia.

### 2.1. Bacterial Isolates

*K. pneumoniae* was recovered from various clinical samples at the microbiology laboratory as part of routine care for hospitalized patients. Clinical samples were cultured on blood agar and MacConkey agar plates (Oxoid Co., Cheshire, England). The isolates were tentatively identified using colony morphology, Gram stain, oxidase, and conventional biochemical tests. Complete identification was confirmed by VITEK II automatic system (bioMerieux, Marcy l’E’toile, France) using the card for Gram-negative strains (ID-GNB) according to the manufacturer’s instructions. *E. coli* ATCC 25922 served as a control strain and was included in each VITEK run testing step. The sampling represented a single collection, and each patient was sampled only once. Infected patients infected with two different bacterial pathogens were excluded from the study. 

### 2.2. Antimicrobial Susceptibility Testing

The antimicrobial susceptibility testing of *K. pneumoniae* was examined using Vitex II cards (AST-N292) (bioMérieux, Marcy l’Etoile, France) according to the manufacturer’s guidelines. The following antimicrobial agents were tested: amikacin (AK), ampicillin (AMP), amoxicillin/clavulanate (AMC), aztreonam (ATM), ceftriaxone (CRO), cefepime (CFPM), cefuroxime (CXM), ciprofloxacin (CIP), trimethoprim/sulfamethoxazole (SXT), colistin (CST), gentamicin (GN), imipenem (IPM), meropenem (MEM), piperacillin/tazobactam (TZP), and tigecycline (TGC) (bioMérieux, Marcy l’Etoile, France). Clinical and Laboratory Standards Institute (CLSI) recommendations were used to determine the minimum inhibitory concentration (MIC) and breakpoints [13]. MDR *K. pneumoniae* was defined as non-susceptibility to at least one agent in three or more antimicrobial categories [14]. *K pneumoniae* ATCC 700603 and *E. coli* ATCC 25922 were used as quality control strains. The isolates were immediately stored in brain heart infusion broth containing 20% glycerol at −86 °C for further genetic analysis. 

### 2.3. Genetic Analysis of MDR Klebsiella pneumoniae Isolates

#### 2.3.1. Detection of Antibiotic Resistance Genes

The *K. pneumoniae* isolates were screened for the carbapenemase genes of *bla*OXA-48-like, *bla*NDM-1, *bla*KPC, *bla*VIM, and *bla*IMP using a multiplex PCR assay, as previously described [15].

#### 2.3.2. DNA Extraction

Deoxyribonucleic acid was extracted from the isolates using the boiling method [16]. In brief, a single pure colony of each strain was mixed in 100 μL sterile free RNA distilled water. The suspension was boiled in a water bath at 95 °C for 10 min, and then the cell debris was precipitated by centrifugation at 15,000 rpm for 5 min. Finally, the supernatant was removed to a new sterile Eppendorf tube and used directly as a template.

DNA template was subjected to multiplex PCR to amplify the target resistance genes. The oligonucleotide primers used in this study are shown in Table 1. The PCR reaction was carried out at a total volume of 50 µL. Each reaction mixture contained 25 µL of HotStarTaq Plus Master Mix (Qiagen GmbH, Hilden, Germany), 4 µL of DNA template, a variable volume of a specific primer group, and 9 µL of nuclease-free water. The Eppendorf Master cycler Gradient instrument was used for the amplification of the target genes with the following optimal cycling conditions: Initial heat activation at 95 °C for 5 min, 35 cycles of denaturation at 94 °C for 45 s, annealing (for *bla*OXA-48-like and *bla*NDM-1 genes optimized at 55 °C for 45 s for *blaI*MP, *bla*VIM, and *bla*KPC genes optimized at 53 °C for 45 s), extension at 72 °C for 1 min and a final extension at 72 °C for 10 min. The amplification products were visualized under ultraviolet illumination at a wavelength of 312 nm after running at 85 volts for 60 min on 2% agarose containing ethidium bromide (1 µg/mL). A 100-bp DNA ladder (Qiagen GmbH, Hilden, Germany) was used as a standard molecular weight to determine the size of the PCR products. DNA from reference *bla*CTX-M, *bla*TEM, and *bla*OXA-like-positive strains was used as a positive control.

### 2.4. Statistical Analysis

The outcome data were entered and analyzed using IBM SPSS version 22.0 (SPSS Inc., Chicago, IL, USA). The descriptive statistics were used in terms of frequency, proportions, means, and standard deviations. The Chi-square test was used to determine the relationships between the presence of the resistance-gene-carrying isolates and the patients’ general and clinical characteristics. 

## 3. Results

### 3.1. Sociodemographic and Clinical Characteristics of Study Participants

A total of 71 *K. pneumoniae* were collected from adult patients with chronic diseases, of whom 38 (53.5%) were males and 33 (46.5%) were females. The mean (±SD) age of patients was 66.15 (±18.6) years, with age ranges from 25 to 44 years (young adults) (37%), 45 to 64 years (35%), and ≥65 years (28%). The isolates were recovered from samples of sputum (25; 35.2%), urine (24; 33.8%), blood (12; 16.9%), wound pus (8; 11.3%), and tracheal aspirate (TIP) (2; 2.8%). Most of the isolates were from the intensive care unit (ICU) (37; 52.1%), medical department (MID) (14; 19.7%), chronic high-dependency unit (CHDU) (9; 12.7%), critical care unit (CCU) (7; 9.9%), urology (4; 5.6%), and emergency (2; 2.8%). Most of the patients have diabetes mellitus (46; 64.8%), hypertension (HTN) (42; 59.2%), being with cerebrovascular accident (CVA) (19; 26.8%), chronic obstructive pulmonary disease (COPD) (17; 23.9%), chronic kidney disease (CKD) (15; 21.1%), heart diseases (14; 19.7%), and cardiovascular diseases (CVD) (4; 5.6%). Of the 71 patients, 23 (32.4%) have single comorbidities, 25 (35.2%) have two comorbidities, 10 (14.1%) have three comorbidities, 11 (15.5%) have four comorbidities, and two (2.8%) have five comorbidities.

### 3.2. Antimicrobial Susceptibility Testing

Table 2 summarizes the rates of the resistance of *K. pneumoniae* to several types of antibiotics. Overall, the highest resistance rate was found for ampicillin (100%), followed by cefuroxime (90.1%), ceftriaxone (85.9%), aztreonam, and trimethoprim/sulfamethoxazole (81.7%, each). Resistance to tigecycline (5.6%) and colistin (12.7%) was less common. The isolates from the ICU revealed the highest resistance rates to the tested antibiotic compared to those isolated from other wards.

### 3.3. Screening of PCR for Resistance Genes

**Figure 1:**
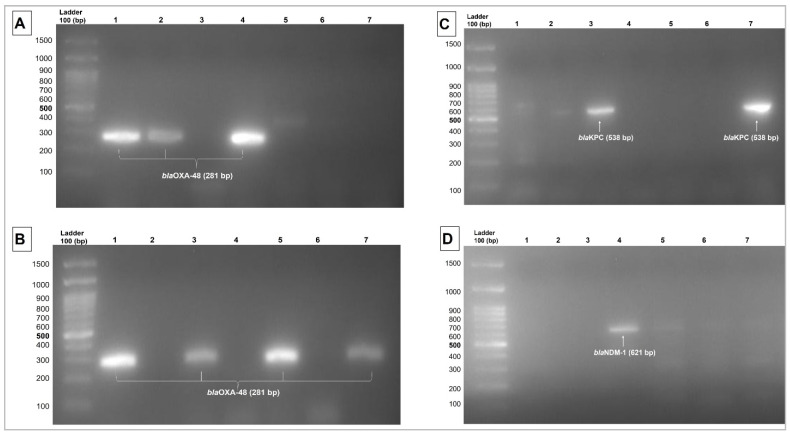
Results of multiplex PCR amplification of genes in MDR *Klebsiella pneumoniae*: (**A**) Lane 1, 2, 4 showed blaOXA-48-like (281 bp) expression; Lane 3, 5, 6, 7 showed negative results. (**B**) lane 1, 3, 5, 7 showed blaOXA-48-like (281bp) expression; Lane 2, 4, 6 were negative. (**C**) Lane 3 and 7 showed blaKPC (538 bp) expression; Lane 1, 2, 4, 5, 6 were negative. (**D**) Lane 4 showed blaNDM-1 (621 bp) expression; Lane 1, 2, 3, 5, 6, 7 were negative.

Of 71 MDR *K. pneumoniae* screened for five resistance genes, 47 (66.2%) isolates harbored various carbapenemase genes (Figure 1). The resistance profile and epidemiological characteristics of the isolate-carrying genes are shown in Supplementary Material Table S1. Most of these 47 isolates were recovered from patients in the ICU (30; 63.8%) followed by medical (7; 14.9%), CHDU (5; 10.6%), CCU (3; 6.4%), and urology (2; 4.3%). The isolates were collected from clinical samples of sputum (17; 36.2%), urine (14; 29.8%), blood (9; 19.1%), wound pus (5; 10.6%), and tracheal aspirate (2; 4.3%). 

**Figure 2:**
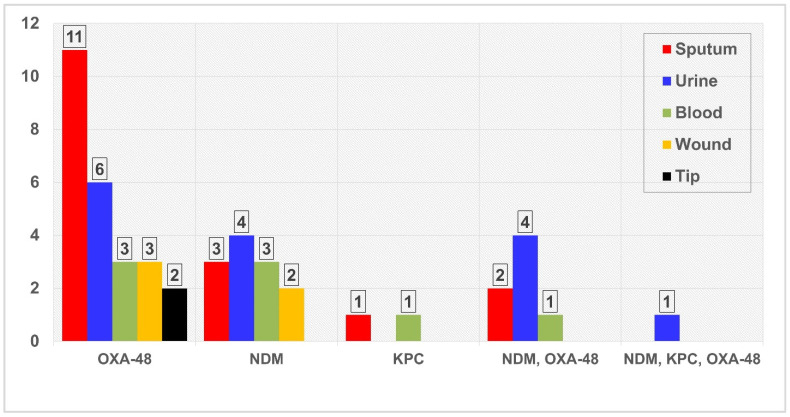
Distribution of carbapenemase-producing MDR. Klebsiella pneumoniae based on the types of clinical specimens.

Figure 2 illustrates the distribution of the resistance genes collected from different clinical specimens. Single resistance genes were found among 40 isolates collected from the clinical specimens. The most prevalent single resistance gene was *bla*OXA-48-like (62.5%; n = 25), and 33.3% of them were recovered from the sputum isolates. *bla*NDM-1 gene was detected in 12(30.0%) isolates, and eight of them were recovered from urine (n = 4) and blood (n = 4). Two (5.0%) single KPC genes were recovered from sputum (n = 1) and blood (n = 1) isolates. In contrast, no *bla*IMP- and *bla*VIM-carrying isolates were detected.

Combinations of two resistance genes between *bla*NDM-1 and *bla*OXA-48-like were found in six strains. Furthermore, combinations of three resistance genes of *bla*NDM-1, *bla*KPC, and *bla*OXA-48-like were found in one isolate. Most of these gene combinations were recovered from urine isolates (Figure 2). 

**Figure 3:**
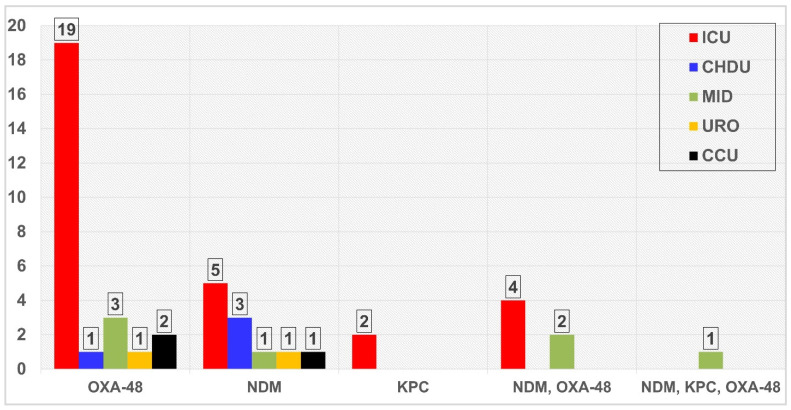
Distribution of carbapenemase-producing MDR. Klebsiella pneumoniae among hospital wards.

Figure 3 illustrates the distribution of resistance genes among hospital wards. Out of the 40 single resistance genes, 26 (65%) were recovered from ICU isolates, namely *bla*OXA-48-like (n = 19), *bla*NDM-1 (n = 5), and *bla*KPC (n = 2). Combinations of two resistance genes were detected in six strains, four from the ICU and two from the medical department. One strain harboring three genes of *bla*NDM-1, *bla*KPC, and *bla*OXA-48-like was recovered from the medical strain.

Table 3 summarizes the frequency of resistance-gene-carrying *K. pneumoniae* among studied patients based on the number of comorbidities. Of the 71 patients with different comorbidities, resistance-gene-carrying isolates were detected in 47 (66.2%) patients. Resistance-gene-carrying isolates were detected in 81.4% of patients with four comorbidities, in 73.9% of patients with single comorbidity, in 70% of patients with three comorbidities, and in 52% of patients with two comorbidities. The types and distribution of comorbidities are shown in Table 3.

### 3.4. Factors Associated with Presence of Resistance Genes

Table 4 shows the analysis of several factors in relation to the tested carbapenemase-gene-carrying *K. pneumoniae* isolates. Generally, the isolates recovered from patients with comorbidities are most likely to have high proportions of antibiotic-resistant genes than those without comorbidities. There were statistically significant associations between the presence of resistance-gene-carrying isolates and gender (χ2(1) = 5.94, *p =* 0.015), ICU admission (χ2(1) = 7.649, *p =* 0.002), CVA (χ2(1) = 6.729, *p =* 0.009), and COPD (χ2(1) = 4.851, *p =* 0.028). There was no statistically significant association between patient age groups, the presence of DM, HTN, CKD, HD, or CVD, the number of comorbidities, and the presence of resistance-gene-carrying isolates.

## 4. Discussion

*K. pneumoniae* is commonly resistant to multiple antibiotics and is considered a source of antimicrobial resistance genes, which can move to other Gram-negative pathogens [9,18]. In the present study, we characterized carbapenemase-producing *K. pneumoniae* and their predicted factors among hospitalized patients with various comorbidities. We applied a multiplex PCR assay to screen the carbapenemase genes of *bla*OXA-48-like, *bla*IMP, *bla*VIM, *bla*KPC, and *bla*NDM-1, which are known as relevant acquired genes commonly identified in clinical *Enterobacteriaceae i*solates worldwide [17]. The present study showed that the production of blaOXA-48-like is the most frequent carbapenemase gene in *K. pneumoniae*. However, most isolates displayed a single carbapenemases gene, of which *bla*OXA-48-like (62.5%) was the most predominant. These results agree with several studies conducted in Saudi Arabia. For instance, a recent study involving thirteen tertiary hospitals across Saudi Arabia found that the overall prevalence of *bla*OXA-48-like carrying *K. pneumoniae* was 71.2%. Furthermore, the proportion of *bla*OXA-48-like was reported at 71% in Asir and 83% in Al Baha, southern Saudi Arabia [19]. Furthermore, in a multicenter study across Saudi Arabia, the *bla*OXA-48-like gene was reported as 69.3% in *K. pneumoniae* and *Escherichia coli* isolated from patients over 14 years old [10]. The predominant *bla*OXA-48-like gene among *K. pneumoniae* clinical isolates has been documented in other studies in Saudi Arabia [6,20,21]. These findings, coupled with our present results, prove the increased proportion of *K. pneumonia* harboring the *bla*OXA-48-like gene in different geographical areas of the country. Likewise, a high frequency of *bla*OXA-48-like among *K. pneumoniae* has been reported in a study of a cross-Gulf cooperation council, including Saudi Arabia. Zowawi et al. indicated that multiple clones of *bla*OXA-48-like- producing *K. pneumoniae* are circulating within hospitals in the Gulf Cooperation Council. This finding suggests that *bla*OXA-48-like producers have been prevalent in hospitals in the region for a prolonged period [20]. 

The present study found that *bla*NDM-1 was the second most frequent carbapenemase gene in our isolates, which agrees with that reported in the country [6,19,20]. Likewise, *bla*NDM-1-carrying *K. pneumoniae* isolates have been reported in neighboring countries such as Yemen [22], Kuwait [23], and the Sultanate of Oman [24]. *bla*NDM-1 gene-carrying strains can harbor multiple chromosomally and plasmid-encoded resistance genes resulting in a multi-drug-resistant trait [5]. Additionally, it has been found that *bla*NDM-1 can inactivate approximately all beat-lactams except aztreonam. Unfortunately, eradicating infections caused by *bla*NDM-1 producers is difficult because treatment options are minimal [25]. The spread of *bla*OXA-48-like- and *bla*NDM-1-carrying isolates in Saudi Arabia hospitals might be due to a high frequency of expatriates and population movement between Saudi Arabia and other parts of the world, such as the Middle East region and the Indian subcontinent [5,26].

We found a low incidence of *bla*KPC genes and an absence of the *bla*VIM and *bla*IMP genes. In a study that examined carbapenemase-producing *K*. *pneumoniae* (n = 60) from hospitalized patients in Saudi Arabia, Shibl et al. found that forty-seven isolates harbored *bla*OXA-48-like, 12 were positive for *bla*NDM, and one for *bla*VIM. No isolate harbored a combination of these resistance genes. In addition, no isolate harbored *bla*KPC or blaIMP [5]. Consistently, another study reported the absence of *bla*VIM, *bla*KPC, and *bla*IMP, suggesting that these genes are not significant sources of carbapenem resistance in Saudi Arabia. This may partly be because these carbapenemase-producing bacteria are common in countries with low population flow to and from Saudi Arabia [6]. 

The co-existence and evolution of antibiotic-resistant genes are one of the most worrying possibilities, as they can lead to the emergence of untreatable invasive *K. pneumoniae* infections [10,27]. In this study, the production of two resistance genes in a single isolate was shared between *bla*OXA-48-like and *bla*NDM-1. This finding is consistent with a previous study in Saudi Arabia, where the co-production of *bla*OXA-48-like and *bla*NDM-1 was detected in *K. pneumoniae* isolated from hospitalized patients in 2012 [6]. A previous study in Riyadh capital, the central region of Saudi Arabia, observed the emergence of MDR *K. pneumoniae* harboring *bla*OXA-48-like and *bla*NDM-1. However, no isolate had a combination of these resistance genes. [5]. In eastern Saudi Arabia, a study conducted in two hospitals found combinations of *bla*OXA-48-like and *bla*NDM-1 with other genes of ESBL resistance, fluoroquinolone resistance, and aminoglycoside resistance among extensively drug-resistant *K. pneumoniae* strains [2]. 

Interestingly, we found the co-existence of triple genes of *bla*NDM-1, *bla*KPC, and *bla*OXA-48-like was detected in one isolate. This is consistent with findings from four tertiary care hospitals in the Makkah region, Saudi Arabia, indicating the emergence of *K. pneumoniae* clinical isolates harboring this combination of carbapenemase-resistant genes [8]. The dissemination and co-evolution of such a co-existence of antibiotic-resistant genes is a health concern, which might lead to untreatable *K. pneumoniae* infections.

The emergence of carbapenemase-producing Gram-negatives is of concern because it is often associated with the occurrence of MDR isolates, which leads to the limitation of therapeutic options [20]. In the present study, the results of antibiotic susceptibility of *K. pneumoniae* isolates are in keeping with the reported MDR phenotype associated with detected carbapenemase genes of *bla*OXA-48-like, *bla*NDM-1, and *bla*KPC. However, the *K. pneumoniae* isolates showed high resistance to various antibiotic groups, including the carbapenem class. Resistance to carbapenem can be due to other mechanisms, such as the production of AmpC and ESBL or structural changes in the outer membrane [6,7]. 

Polymyxins and tigecycline are treatment options for carbapenem-resistant *K. pneumoniae* infections, but their use is limited by concerns about their efficacy and safety [28]. *K. pneumoniae* isolates from our hospital showed a worrying increase in tigecycline and colistin resistance, which are the last effective drugs of choice [29]. This is in line with a study carried out in three hospitals in Riyadh, where the rate of tigecycline resistance to *K. pneumoniae* obtained from hospitalized patients was 4.8% [21]. In addition, resistance to colistin has been observed among *K. pneumoniae* isolates from inpatients in Riyadh city [5]. Similarly, the proportion of resistance to tigecycline(12.5%) was determined among *K. pneumoniae* collected from patients in two hospitals in the eastern Province of Saudi Arabia [2]. The occurrence of carbapenemase-producing bacteria and increasing their resistance to colistin and tigecycline might challenge public health. Therefore, implementing effective control strategies to combat the spreading of carbapenemase producers is needed. 

Identifying risk factors for carbapenemase-producing bacterial pathogens is critical for selecting appropriate therapy and maintaining effective control and prevention plans [12,30]. The present finding indicates that ICU patients were at risk of acquiring infection with *K. pneumoniae* carrying carbapenemase during their course of admission to the ICU. ICU admission has been observed as a predictor of infection with carbapenemase-producing *K. pneumoniae* [31,32] and the emergence and spread of MDR bacteria in many studies [27,33,34,35]. The high prevalence of carbapenem-resistant *K. pneumoniae* in ICU may be related to the heavy use of invasive procedures, the immune suppressant condition of ICU patients, the presence of multiple infections in patients, and the extensive usage of high-grade antimicrobials [34]. To reduce the risk of spreading MDR pathogens, infection prevention strategies and screening the patients for carbapenemase and other resistance-determinant-carrying bacteria should be designed during ICU admission. 

In the present study, patients with comorbidities are most likely to acquire carbapenem-resistance-gene-carrying *K. pneumoniae*. A systematic review and meta-analysis of mortality of patients with comorbidities associated with carbapenem-resistant *K. pneumoniae* infections found a high proportion of mortality in patients with chronic kidney disease, diabetes, and liver disease. However, the risk of death from carbapenem-resistant *K. pneumoniae* was twice as high as the number of deaths associated with carbapenem-susceptible *K. pneumoniae* (*p* < 0.00001) [11]. Patients with comorbidities are usually immune compromised, are usually undergoing invasive medical procedures, tend to be prolonged hospital stays, and are exposed to different types of antibiotics, which increase the opportunity to acquire infection with carbapenem-resistant *K. pneumoniae* [11]. On the other hand, our findings showed that patients with chronic obstructive pulmonary infections were significantly at high risk of acquiring carbapenemase-producing *K. pneumoniae*. Research evidence indicates that respiratory tract infection is the most common infection among all infections among hospitalized patients [11]. It is well known that hospitalized patients with critical respiratory tract infections are usually under invasive intubation procedures and at high risk of the exogenous acquisition of antibiotic-resistant bacteria through direct and indirect contact, which might be factors for the spread of carbapenemase genes [34]. Our result did not find a significant association between the acquisition of carbapenemase-gene-carrying *K. pneumoniae* and the number of comorbidities; however, these three or more comorbidities were found to be an independent risk factor for carbapenem-resistant *K. pneumoniae* infections in a recent study [11].

### Limitations of the Study

Our study had several limitations: First, this study was a single-center study. Second, we used small numbers of isolates to analyze limited numbers of predicted factors of carbapenemase producers. A systematic review and meta-analysis showed that eighteen factors might increase the risk of carbapenem resistance in *K. pneumoniae* infection [36]. However, a survey on a large scale using multivariate analysis might show a clear correlation between the presence of carbapenemase producers and other patients’ clinical characteristics, such as admission outcomes, antibiotics use, and hospitalization history. Third, we characterized only clinical MDR strains collected from patients with comorbidity to evaluate carbapenemase production, which might generate a selection bias. Fourth, the study analyzed the major resistance determinant of carbapenem; other minor carbapenemase genes of *bla*GIM, *bla*SIM, *bla*AIM, *bla*DIM, and *bla*BIC were not investigated to achieve a better evaluation of actual prevalence. 

## 5. Conclusions

The study highlighted the existence of carbapenemase-producing *K. pneumoniae*, particularly *bla*OXA-48-like and *bla*NDM-1, in patients with comorbidities in our hospital setting. Therefore, the early detection of carbapenemase producers, tracking the source of resistant isolates, and isolation of patients at risk of carbapenemase carriage are important. Our findings emphasize the importance of the routine implementation of molecular characterization assays in the hospital laboratory for the appropriate detection of resistance-determinant-carrying bacterial pathogens and a better understanding of the mechanism of antibiotic resistance. The study will be a valuable reference for further research to identify the predictor factors of acquisition carbapenemase-producing microorganisms. However, regression analysis with large sample size is needed to obtain accurate findings to this association.

## Figures and Tables

**Table 1:** Oligonucleotides used in this study.

Gene	Primers	Sequence	Band/bp	Reference
*bla*_NDM-1_	NDM-F NDM-R	GGTTTGGCGATCTGGTTTTC CGGAATGGCTCATCACGATC	621	[17]
*bla*_IMP_	IMP-F IMP-R	TTGACACTCCATTTACDG GATYGAGAATTAAGCCACYCT	139	[15]
*bla*_VIM_	VIM-F VIM-R	GATGGTGTTTGGTCGCATA CGAATGCGCAGCACCAG	390	[15]
bla_KPC_	KPC-F KPC-R	CATTCAAGGGCTTTCTTGCTGC ACGACGGCATAGTCATTTGC	538	[15]
*bla*_OXA-48-like_	OXA-F OXA-R	GCTTGATCGCCCTCGATT GATTTGCTCCGTGGCCGAAA	281	[15]

**Table 2:** Antibiotic resistance rates of MDR *Klebsiella pneumoniae* (n = 71) collected from comorbidity patients at different hospital’ wards.

Agent	Overall, n (%)	ICU, n (%)	Medical, n (%)	CCU, n (%)	CHDU, n (%)	Urology, n (%)
Amikacin	43 (60.6)	28 (75.7)	8 (57.1)	2 (28.6)	4 (44.4)	1 (25)
Ampicillin	71 (100)	(100)	(100)	(100)	(100)	(100)
Amoxicillin/clavulanate	54(76.1)	33 (89.2)	10 (71.4)	4 (57.1)	5 (55.6)	2 (50)
Aztreonam	58 (81.7)	34 (91.9)	12 (85.7)	4 (57.1)	6 (66.7)	2 (50)
Ceftriaxone	61 (85.9)	36 (97.3)	11 (78.6)	4 (57.1)	7 (77.8)	3 (75)
Cefepime	57 (80.3)	35 (94.6)	9 (64.3)	4 (57.1)	6 (66.7)	3 (75)
Cefuroxime	64 (90.1)	36 (97.3)	13 (92.9)	5 (71.4)	7 (77.8)	3 (75)
Ciprofloxacin	49 (69)	30 (81.1)	8 (57.1)	4 (57.1)	6 (66.7)	1 (25)
trimethoprim/sulfamethoxazole	58 (81.7)	32 (86.5)	11 (78.6)	6 (85.7)	6 (66.7)	3 (75)
Colistin	9 (12.7)	9 (24.3)	0 (0.0)	0 (0.0)	0 (0.0)	0 (0.0)
Gentamicin	45(63.4)	29 (78.4)	7 (50)	3 (42.9)	4 (44.4)	2 (50)
Imipenem	40 (56.3)	29 (78.4)	4 (28.6)	3 (42.9)	3 (33.3)	1 (25)
Meropenem	49 (69)	33 (89.2)	6 (42.9)	2 (28.6)	6 (66.7)	2 (50)
Piperacillin/tazobactam	57 (80.3)	35 (94.6)	9 (64.3)	4 (57.1)	6(66.7)	3 (75)
Tigecycline	4 (5.6)	3 (8.1)	1 (7.1)	0 0.0 (0.0)	0 0.0 (0.0)	0 0.0 (0.0)

**Table 3:** Distribution of resistance-gene-carrying *Klebsiella pneumoniae* among studied patients based on the number of comorbidities.

Comorbidity	Number of Patients	Frequency of Resistance-Gene-Carrying *K. pneumoniae*, n (%)
**Single**	**23**	**17(73.9)**
COPD	8	7 (87.5)
DM	7	4 (57.1)
HTN	5	3 (60)
CVA	2	2 (100)
HD	1	1 (100)
**Two**	**25**	**13 (52)**
DM, HTN	8	5 (62.5)
DM, CVA	4	0 (0.0)
DM, COPD	3	3 (100)
HTN, COPD	3	3 (100)
HD, CVA	3	1 (33.3)
HTN, CVA	2	00.0
DM, CKD	1	00.0
HTN, CKD	1	1 (100)
**Three**	**10**	**7 (70)**
DM, HTN, CKD	4	3 (75)
DM, HTN, CVA	4	2 (50)
DM, HTN, CVD	1	1 (100)
DM, HTN, HD	1	1 (100)
**Four**	**11**	**9 (81.8)**
DM, HTN, CKD, HD	5	4(80)
DM, HTN, HD, CVA	2	2 (100)
DM, HTN, CKD, COPD	1	1 (100)
DM, HTN, HD, COPD	1	0 (0.0)
DM, HTN, CKD, CVD	1	1 (100)
DM, HTN, CVD, CVA	1	1 (100)
**Five**	**2**	**1 (50)**
DM, HTN, CKD, HD, COPD	1	1 (100)
DM, HTN, CKD, CVD, CVA	1	00.0

CKD, chronic kidney diseases; COPD, chronic obstructive pulmonary diseases; CVA, cerebrovascular accident; CVD, cardiovascular diseases; DM, diabetes mellitus; HD, heart diseases; HTN, hypertension.

**Table 4:** Factors associated with the presence of resistance-gene-carrying *Klebsiella pneumoniae*.

Variable	Total	n (%) of Resistant Gene	χ2	*p* Value
Gender			5.94	0.015
Male	38	30 (78.9)		
Female	33	17 (51.5)		
Age group, year			1.706	0.426
≤45	11	9 (81.8)		
46 to 65	16	11 (68.8)		
>65	44	27 (61.4)		
ICU admission			7.649	0.002
Yes	37	30 (81.1)		
No	34	17 (50)		
Diabetes Mellitus			0.446	0.581
Yes	46	29(63)		
No	25	18 (72)		
Hypertension			0.541	0.373
Yes	42	29(69)		
No	29	18 (62.1)		
Chronic kidney diseases			0.433	0.511
Yes	15	11 (73.3)		
No	56	36 (64.3)		
Heart diseases			0.213	0.644
Yes	14	10 (71.4)		
No	57	37 (64.9)		
Cardiovascular diseases			0.147	0.702
Yes	4	3 (75)		
No	67	44 (65.7)		
Cerebrovascular accident			6.729	0.009
Yes	19	8 (42.1)		
No	52	39 (75)		
Chronic obstructive pulmonary diseases			4.851	0.028
Yes	17	15 (88.2)		
No	54	32(59.3)		
Number of comorbidities			3.476	0.176
One	23	17(73.9)		
Two	25	13 (52)		
Three or more	23	17(73.9)		

## Data Availability

The raw data of the study are available upon reasonable request. All the data generated or analyzed during the study are included in this manuscript.

## Supplementary Materials

The following supporting information can be downloaded at: https://www.mdpi.com/article/10.3390/antibiotics11121697/s1, Table S1: Epidemiological characteristics and resistant profiles of carbapenemase resistant genes carrying *K. pneumoniae* recovered from adult patients.
