# Supplementary material for: Molecular Epidemiology of Carbapenem-Resistant K. pneumoniae Clinical Isolates from the Adult Patients with Comorbidities in a Tertiary Hospital, Southern Saudi Arabia
Source: Antibiotics (Basel). 2022 Nov 25;11(12):1697. doi: 10.3390/antibiotics11121697 (PMC9774885; doi:10.3390/antibiotics11121697)
Supplement: Supplementary file 1 [file antibiotics-11-01697-s001.zip › antibiotics-2029881-supplementary.pdf]

**Table S1.** Epidemiological characteristics and resistant profiles of carbapenemase resistant genes carrying *K. pneumoniae* recovered from adult patients.

| N0 | Sex | Sample | Ward* | Resistant Gene | No. of Resistant Isolates | Resistant Profile** |
|---|---|---|---|---|---|---|
| 1. | M | Sputum | CHDU | OXA-48 | 7 | AMP, ATM, CRO, CIP, GN, IPM, MEM |
| 2. | M | Sputum | ICU | OXA-48 | 7 | AMP, CRO, CFPM, CXM, IPM, MEM, TZP |
| 3. | F | Sputum | CHDU | OXA-48 | 12 | AK, AMP, AMC, ATM, CRO, CFPM, CXM, CIP, SXT, GN, MEM TZP |
| 4. | M | Urine | ICU | OXA-48 | 12 | AK, AMP, AMC, ATM, CRO, CFPM, CXM, CIP, SXT, GN, MEM, TZP |
| 5. | M | Blood | ICU | OXA-48 | 12 | AK, AMP, AMC, ATM, CRO, CFPM, CXM, CIP, SXT, GN, MEM, TZP |
| 6. | M | Sputum | ICU | OXA-48 | 12 | AK, AMP, AMC, ATM, CRO, CFPM, CXM, CIP, SXT, GN, MEM, TZP |
| 7. | M | Sputum | ICU | OXA-48 | 12 | AMP, AMC, ATM, CRO, CFPM, CXM, CIP, SXT, CST, IPM, MEM, TZP |
| 8. | M | Urine | ICU | OXA-48 | 12 | AK, AMP, AMC, ATM, CRO, CFPM, CXM, CIP, SXT, IPM MEM, TZP |
| 9. | M | Urine | ICU | OXA-48 | 12 | AK, AMP, AMC, ATM, CRO, CFPM, CXM, CIP, SXT, GN, MEM, TZP |
| 10. | F | Urine | ICU | OXA-48 | 12 | AK, AMP, AMC, ATM, CRO, CFPM, CXM, CIP, SXT, IPM, MEM, TZP |
| 11. | F | Blood | MID | OXA-48 | 12 | AK, AMP, AMC, ATM, CRO, CFPM, CXM, CIP, SXT, GN, MEM, TZP |
| 12. | F | Blood | MID | OXA-48 | 12 | AK, AMP, AMC, ATM, CRO, CFPM, CXM, CIP, SXT, GN, MEM, TZP |
| 13. | M | Sputum | CCU | OXA-48 | 13 | AK, AMP, AMC, ATM, CRO, CFPM, CXM, CIP, SXT, GN, IPM, MEM, TZP |
| 14. | M | Sputum | CCU | OXA-48 | 13 | AK, AMP, AMC, ATM, CRO, CFPM, CXM, CIP, SXT, GN, IPM, MEM, TZP |
| 15. | M | Sputum | ICU | OXA-48 | 13 | AK, AMP, AMC, ATM, CRO, CFPM, CXM, CIP, SXT, GN, IPM, MEM, TZP |
| 16. | F | Wound | ICU | OXA-48 | 13 | AK, AMP, AMC, ATM, CRO, CFPM, CXM, CIP, SXT, GN, IPM, MEM, TZP |
| 17. | M | Sputum | ICU | OXA-48 | 13 | AK, AMP, AMC, ATM, CRO, CFPM, CXM, CIP, SXT, GN, IPM, MEM, TZP |
| 18. | M | Sputum | ICU | OXA-48 | 13 | AK, AMP AMC, ATM, CRO, CFPM, CXM, CIP, SXT, GN, IPM, MEM, TZP |
| 19. | M | Wound | ICU | OXA-48 | 13 | AK, AMP, AMC, ATM, CRO, CFPM, CXM, CIP, SXT, GN, IPM, MEM, TZP |
| 20. | M | Wound | MID | OXA-48 | 13 | AK, AMP, AMC, ATM, CRO, CFPM, CXM, CIP, SXT, GN, IPM, MEM, TZP |
| 21. | F | TIP | ICU | OXA-48 | 14 | AK, AMP, AMC, ATM, CRO, CFPM, CXM, CIP, SXT, CST, GN, IPM, MEM, TZP |
| 22. | F | TIP | ICU | OXA-48 | 14 | AK, AMP, AMC, ATM, CRO, CFPM, CXM, CIP, SXT, CST, GN, IPM, MEM, TZP |
| 23. | M | Urine | ICU | OXA-48 | 14 | AK, AMP, AMC, ATM, CRO, CFPM, CXM, CIP, SXT, CST, GN, IPM, MEM, TZP |
| 24. | M | Urine | ICU | OXA-48 | 15 | AK, AMP, AMC, ATM, CRO, CFPM, CXM, CIP, SXT, CST, GN, IPM, MEM, TZP, TGC |
| 25. | F | Sputum | ICU | OXA-48 | 15 | AK, AMP, AMC, ATM, CRO, CFPM, CXM, CIP, SXT, GN, IPM, MEM, TZP |
| 26. | F | Blood | ICU | OXA-48 | 15 | AK, AMP, AMC, ATM, CRO, CFPM, CXM, CIP, SXT, CST, GN, IPM, MEM, TZP, TGC |
| 27. | M | Urine | URO | NDM-1 | 7 | AMP, CRO, CFPM, CXM, GN, MEM, TZP |
| 28. | M | Urine | CHDU | NDM-1 | 8 | AMP, CRO, CFPM, CXM, SXT, IPM, MEM, TZP |
| 29. | M | Blood | CHDU | NDM-1 | 9 | AMP, AMC, ATM, CRO, CFPM, CXM, IPM, MEM, TZP |
| 30. | M | Blood | ICU | NDM-1 | 9 | AMP, AMC, ATM, CRO, CFPM, CXM, IMP, MEM, TZP |
| 31. | M | Sputum | CCU | NDM-1 | 9 | AMP, AMC, ATM, CRO, CFPM, CXM, IPM, MEM, TZP |
| 32. | F | Urine | ICU | NDM-1 | 11 | AMP, AMC, ATM, CRO, CFPM, CXM, SXT, GN, MEM, IMP, TZP |

| | | | | | | |
|---|---|---|---|---|---|---|
| 33. | F | Wound | ICU | NDM-1 | 11 | AMP, AMC, ATM, CRO, CFPM, CXM, CIP, TZP, IPM, MEM, TZP |
| 34. | F | Wound | MID | NDM-1 | 10 | AMP, ATM, CRO, CFPM, CXM, SXT, GN, IPM, MEM, TZP |
| 35. | F | Blood | CHDU | NDM-1 | 11 | AK, AMP, AMC, ATM, CRO, CFPM, CXM, CIP, GN, MEM, TZP |
| 36. | M | Sputum | ICU | NDM-1 | 12 | AK, AMP, AMC, ATM, CRO, CFPM, CXM, SXT, GN, IPM, MEM, TZP |
| 37. | F | Sputum | ICU | NDM-1 | 13 | AK, AMP, AMC, ATM, CRO, CFPM, CXM, CIP, SXT, GN, IPM, MEM, TZP |
| 38. | M | Urine | URO | NDM-1 | 13 | AK, AMP, AMC, ATM, CRO, CFPM, CXM, CIP, SXT, GN, IPM, MEM, TZP |
| 39. | F | Blood | ICU | KPC | 14 | AK, AMP, AMC, ATM, CRO, CFPM, CXM, CIP, SXT, CST, GN, IPM, MEM, TZP |
| 40. | F | Sputum | ICU | KPC | 15 | AK, AMP, AMC, ATM, CRO, CFPM, CXM, CIP, SXT, CST, GN, IPM, MEM, TZP, TGC |
| 41. | M | Urine | MID | NDM-1, OXA-48 | 9 | AK, AMP, AMC, ATM, CRO, CFPM, CXM, CIP, SXT |
| 42. | M | Blood | ICU | NDM-1, OXA-48 | 12 | AK, AMP, AMC, ATM, CRO, CXM, CIP, SXT, GN, IPM, MEM, TZP |
| 43. | M | Sputum | ICU | NDM-1, OXA-48 | 12 | AMP, AMC, ATM, CRO, CFPM, CXM, CIP, SXT, GN, IPM, MEM, TZP |
| 44. | M | Urine | ICU | NDM-1, OXA-48 | 13 | AK, AMP, AMC, ATM, CRO, CFPM, CXM, CIP, SXT, GN, IPM, MEM, TZP |
| 45. | F | Sputum | ICU | NDM-1, OXA-48 | 14 | AK, AMP, AMC, ATM, CRO, CFPM, CXM, CIP, SXT, CST, GN, IPM, MEM, TZP |
| 46. | M | Urine | MID | NDM-1, OXA-48 | 14 | AK, AMP, AMC, ATM, CRO, CFPM, CXM, CIP, SXT, GN, IPM, MEM, TZP, TGC |
| 47. | M | Urine | MID | NDM, KPC, OXA-48 | 13 | AK, AMP, AMC, ATM, CRO, CFPM, CXM, CIP, SXT, GN, IPM, MEM, TZP |

*CCU critical care unit, CHDU chronic high dependency unit, CKD chronic kidney disease, COPD chronic obstructive pulmonary disease, CVA cerebrovascular accident, CVD cardiovascular diseases, DM diabetes mellitus, HD heart diseases, HTN hypertension, MID medical department, TIP tracheal aspirate, UR urology. ** AK amikacin, AMP ampicillin, AMC amoxicillin/clavulanate, ATM aztreonam, CRO ceftriaxone, CFPM cefepime, CXM cefuroxime, CIP ciprofloxacin, SXT trimethoprim/sulfamethoxazole, CTS colistin, GN gentamicin, IPM imipenem, MEM meropenem, TZP piperacillin/tazobactam, TGC tigecycline
